# CT-Based Software-Generated Measurements Permit More Objective Assessments of Arithmetic Hip-Knee-Ankle Axis and Joint Line Obliquity

**DOI:** 10.3390/life15020188

**Published:** 2025-01-27

**Authors:** Wai Kit Wong, Siti Zubaidah Zulkhairi, Hwa Sen Chua

**Affiliations:** 1 Department of Orthopaedics and Traumatology, Hospital Ampang, Ampang 68000, Selangor, Malaysia; 2 Clinical Research Centre, Sunway Medical Centre, Bandar Sunway, Subang Jaya 47500, Selangor, Malaysia; szubaidahz@sunway.com.my; 3 Orthopaedic Centre of Excellence, Sunway Medical Centre, Bandar Sunway, Subang Jaya 47500, Selangor, Malaysia; hwasen@gmail.com

**Keywords:** constitutional alignment, arithmetic hip–knee–ankle axis (aHKA), robotic-assisted total knee arthroplasty, Mako

## Abstract

The rapid adoption of robotic-assisted total knee arthroplasty (RATKA) has resulted in pre-operative CT scans becoming more readily available. After the segmentation and identification of landmarks by trained segmentation specialists, the Mako SmartRobotics^TM^ software generates measurements of interest for the calculation of the arithmetic hip-knee-ankle axis (aHKA), joint line obliquity (JLO), and the Coronal Plane Alignment of the Knee (CPAK) phenotype. The aim of this study is to ascertain how closely correlated these two sets of readings are and whether the CPAK distribution is altered when comparing both modalities. A retrospective radiological study was undertaken on 500 knees (367 patients: 133 bilateral, 234 unilateral) comparing the CT-based software-generated measurements of patients undergoing RATKA using the Stryker Mako system against manual measurements derived from long limb radiographs (LLRs). There were statistically significant differences between the average measurements of the LDFA (0.27 ± 2.95, *p* = 0.045), MPTA (1.15 ± 2.20, *p* < 0.001), aHKA (1.41 ± 3.85, *p* < 0.001) and JLO (0.89 ± 3.50, *p* < 0.001), with CT measurements having higher mean readings for LDFA, lower readings for MPTA, more varus aHKA and increased apex distal JLO. Despite this, correlation was moderately good: LDFA (*r* = 0.409, *p* < 0.001), MPTA (*r* = 0.683, *p* < 0.001), aHKA (*r* = 0.595, *p* < 0.001) and JLO (*r* = 0.456, *p* < 0.001). The CPAK distribution was also significantly different. LLRs underestimate the degree of constitutional varus and JLO compared to CT-based software-generated measurements, with a resultant increase in CPAK Types I and IV when using CT measurements. Despite moderately good correlation between both imaging modalities, there remains a statistically significant difference between them.

## 1. Introduction

Coronal alignment restoration is a key component of successful total knee arthroplasty (TKA) [1]. Dissatisfaction rates remain substantially high despite good survivorship data and advancements in implant designs [2,3]. Attempts at improving patient satisfaction have led to the realization that there is significant variability in the constitutional alignment of patients. Bellemans first introduced the concept of constitutional varus, reporting that 32% of men and 17% of women in their study cohort had an alignment of 3° varus or more [4]. MacDessi recently introduced the Coronal Plane Alignment of the Knee (CPAK) classification utilizing long limb radiographs to measure the lateral distal femoral angle (LDFA) and medial proximal tibial angle (MPTA) [5]. Instead of the traditional mechanical hip–knee–ankle axis (mHKA), the team utilized an arithmetic method to obtain the arithmetic hip–knee–ankle axis (aHKA = MPTA − LDFA), which ignores the joint line convergence angle (JLCA) of approximately 0.5° [6]. The aHKA has been reported to be representative of constitutional alignment [7]. As the aHKA is derived using only bony landmarks, it is not affected by the spatial relationship between the femur and tibia. As such, the measurements are not influenced by joint space narrowing, ligamentous laxity or tibiofemoral subluxation [6]. The CPAK classification also considers another important parameter: joint line obliquity (JLO = MPTA + LDFA). Both the aHKA and JLO are then utilized to assign patients to one of nine distinct phenotypes [5].

**Figure 1:**
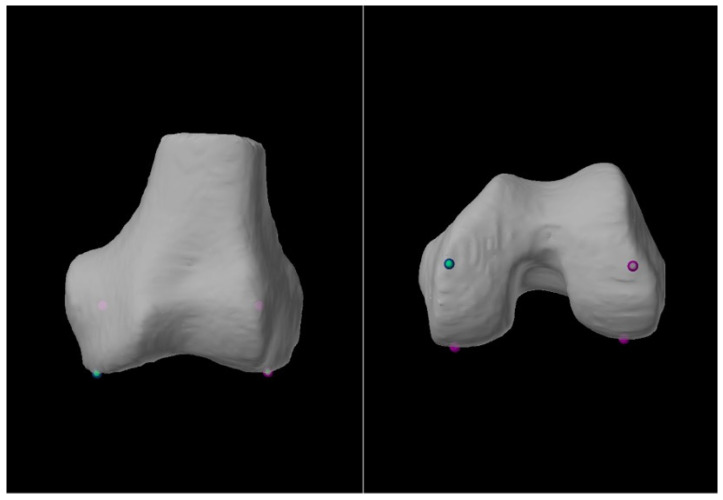
Constitutional landmark points on the distal femur as determined by the software, visualized as green spheres.

**Figure 2:**
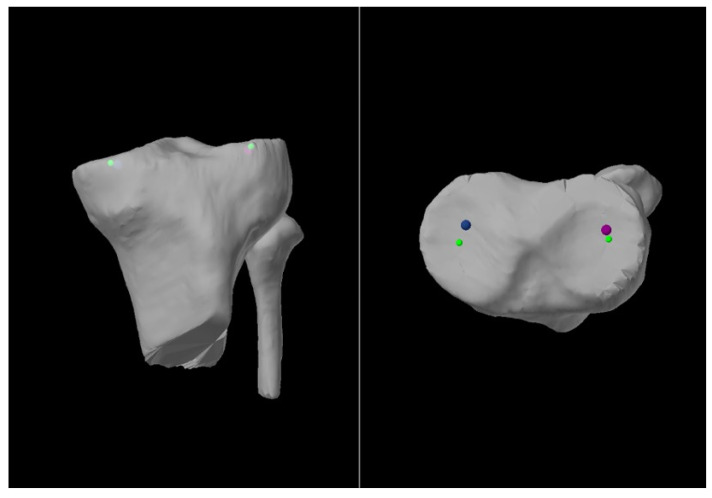
Constitutional landmark points on the proximal tibia as determined by the software, visualized as green spheres.

Traditionally, weight-bearing long limb radiographs (LLRs) have been the gold standard imaging modality for calculating the HKA axis and guiding alignment correction. Robotic systems for arthroplasty can be imageless or image-based, with some image-based systems mandating a pre-operative CT scan for accurately planning bone cuts to facilitate precise implant positioning. Numerous studies have reported comparisons between using LLRs and CT scans for obtaining coronal alignment measures with results varying between the studies [8,9,10,11]. In all these studies, the measurements were performed by members of the surgical team, and human error is unavoidable despite researchers’ best attempts to minimize this. There has recently been an introduction of accompanying software that leverages technology to perform these measurements, which were previously carried out by humans. Upon the successful upload and segmentation of the pre-operative CT scan, the necessary bony anatomical points are identified by trained segmentation specialists. The Mako SmartRobotics^TM^ System (Stryker US (Mako Surgical Corp), Fort Lauderdale, FL, USA) software (Mako TKA 2.0) then generates the alignment measurements of interest. These are better depicted in Figure 1 and Figure 2 below.

**Figure 3:**
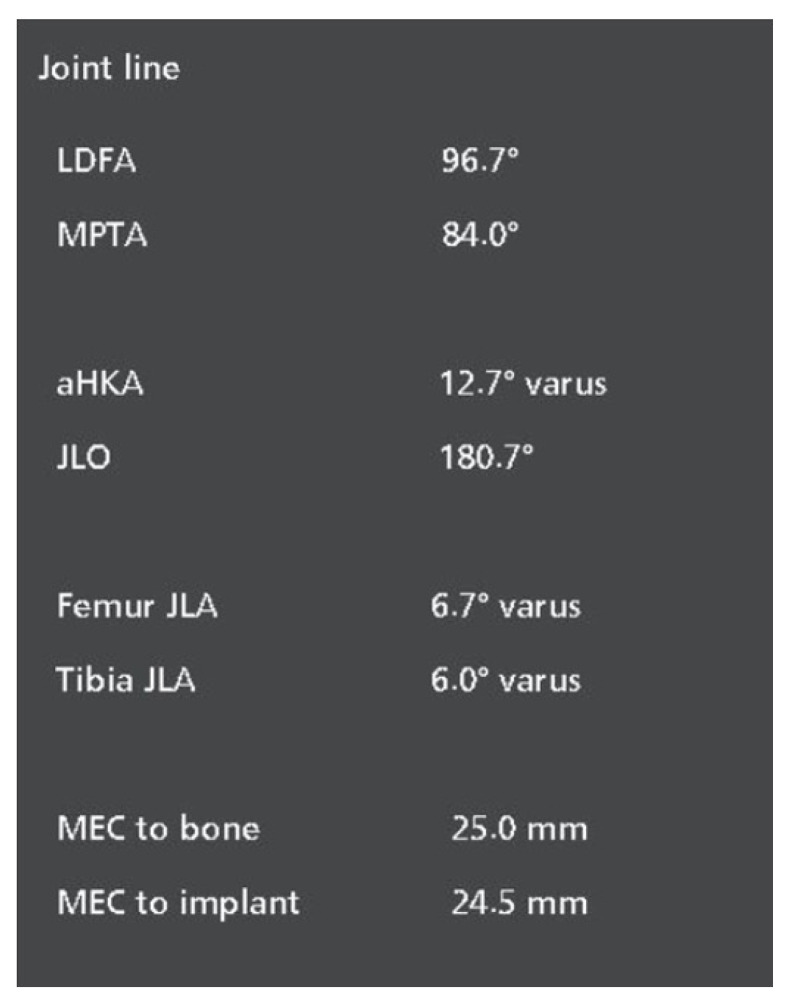
Software-generated measurements available to the surgeon upon the successful upload, segmentation and verification of the pre-operative CT scan.

The software then generates the values of parameters such as the LDFA, MPTA, and the resultant aHKA, as well as the JLO, thus minimizing the bias of human error. A screen capture of what appears on the Mako robot is depicted in Figure 3 below.

To our knowledge, there has not been any report comparing software-generated measurements to conventional human measurements. Our primary objective is to ascertain how closely correlated CT-based software-generated measurements are compared to conventional human measurements based on LLRs, with a secondary aim of assessing whether there are significant differences from the CPAK distribution when utilizing both methods of measurement. The hypothesis posits that software-generated and human measurements correlate well and that the CPAK distribution is not significantly different when comparing both methods of measurement.

## 2. Materials and Methods

### 2.1. Patients

**Figure 4:**
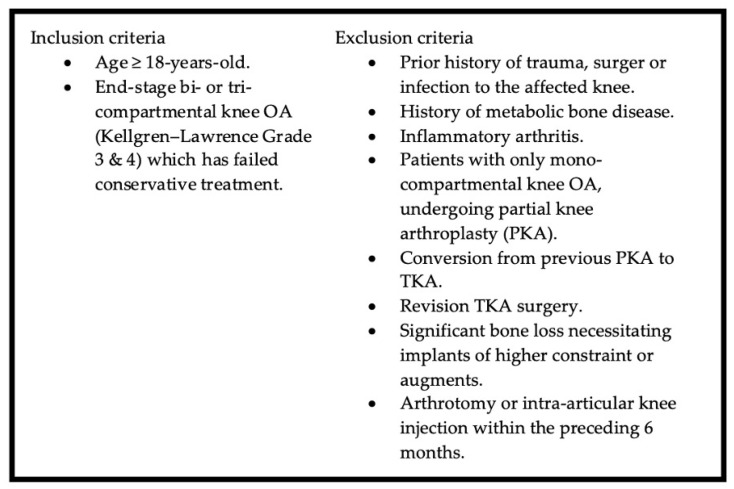
Inclusion and exclusion criteria for enrolment into the study.

A retrospective review of all the patients who underwent a robotic-assisted TKA using the Robotic Arm Interactive Orthopedic System (RIO; MAKO Stryker, Fort Lauderdale, FL, USA) was carried out after obtaining ethical clearance from Sunway Medical Centre Independent Research Ethics Committee (SREC No.: 044/2024/IND/ER). The first 500 knees that fulfilled the inclusion and exclusion criteria as listed in Figure 4 below were included in this study.

### 2.2. Radiological Assessment

At our center, all patients are routinely subjected to long limb radiographs as part of preoperative planning. These were carried out in accordance with the established protocol by Paley [12]. Radiographic images were stored on a PACS system, and all alignment parameters measured using the built-in measurement tools.

The lateral distal femoral angle (LDFA) is defined as the lateral angle formed between the mechanical axis of the femur and the joint line of the distal femur. The medial proximal tibial angle (MPTA) is defined as the medial angle formed between the mechanical axis of the tibia and the joint line of the proximal tibia [5].

The mechanical axis of the femur is defined by a line connecting the center of the femoral head and the center of the knee, and the mechanical axis of the tibia is represented by a line connecting the center of the knee and the center of the ankle. The center of the femoral head is identified via the concentric-circle method, and the center of the ankle is defined by the midpoint of the talus [13]. As per the reference study by MacDessi et al., the constitutional alignment of the patient is approximated by the “arithmetic HKA, aHKA,” which is obtained using the following formula: aHKA = MPTA − LDFA [7]. A negative value equates to a varus alignment, and a positive value denotes valgus. Neutral alignment is limited to 0 ± 2°, inclusive [5].

The second important parameter in the determination of the CPAK classification, the joint line obliquity (JLO), is calculated according to the formula JLO = MPTA + LDFA [5]. The degree of obliquity is measured in relation to the floor with both feet planted in a double-leg stance. The JLO is considered neutral or parallel to the horizontal if JLO = 180 ± 3°, inclusive. A value of ≤176.9° denotes an apex-distal joint line, whereas a JLO of ≥183.1° is apex-proximal [5].

**Figure 5:**
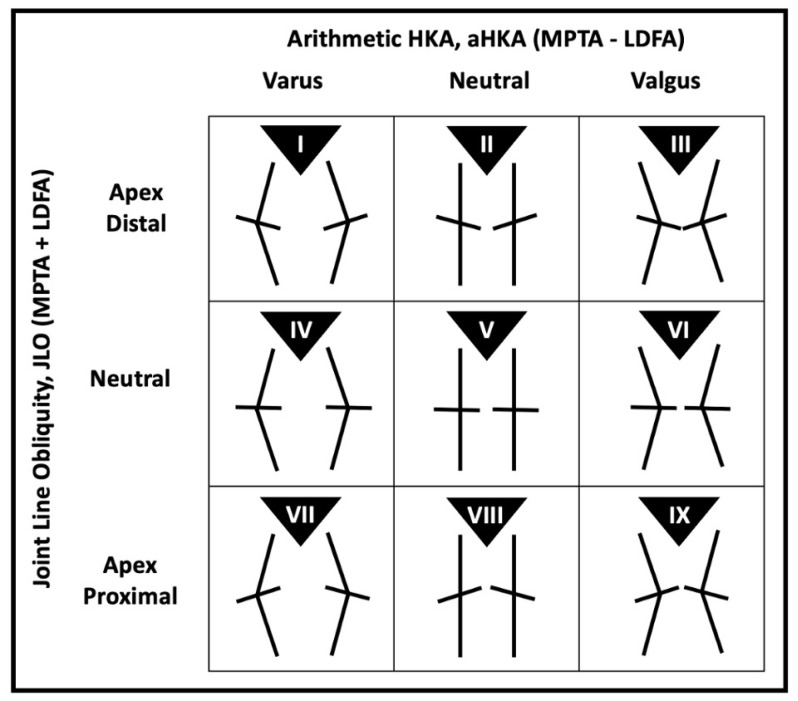
Coronal Plane Alignment of the Knee (CPAK) classification.

Upon determination of the aHKA and JLO, the patients are then assigned a CPAK phenotype, as depicted below in Figure 5.

The following parameters are then extracted from the Mako SmartRobotics^TM^ System software: LDFA, MPTA, aHKA and JLO. The CPAK phenotype was then manually assigned.

### 2.3. Data Analysis

Statistical analyses were performed using SPSS Statistics v.27 (IBM, Armonk, NY, USA). Descriptive statistics were reported as the mean ± standard deviation (x ± s). Normality was assessed with the Shapiro–Wilk test and Q–Q plots. Paired sample *t*-tests and Pearson correlation coefficients were used to compare measurement differences and the relationship strength between the LLR and CT techniques. Frequencies between LLR and CT within CPAK types for each JLO class were compared using a chi-squared test in a 3 × 2 contingency table, while Fisher’s exact test was used for combined-group comparisons in a 2 × 2 table for distal and neutral JLO. The statistical significance was set at *p* < 0.05. The intraclass correlation coefficient was utilized to ascertain the intra- and inter-observer reproducibility of measurements on a randomly generated subgroup of 30 knees. The measurements were performed separately by a senior surgeon (CHS), who is a fellowship-certified arthroplasty surgeon, an orthopedic surgeon (WWK) and a trainee doctor. The measurements were repeated at one-week intervals for a further two sets of readings.

## 3. Results

### 3.1. Demographic Data

In this study, there were 337 females (67.4%) and 163 males (32.6%). The mean age was 66.53 years (range: 47–88). There were 278 right (55.6%) and 222 left (44.4%) knees. The average BMI was 27.39 kg/m^2^.

### 3.2. Primary Outcome

There were statistically significant differences between LLRs and CT-based software-generated values for the average measurements of the LDFA, MPTA, aHKA and JLO. Using Cohen’s *d* to determine effect size, LDFA, aHKA and JLO had small effect sizes, but MPTA had a moderate effect size. These are captured in detail in Table 1 below. Despite the significant differences between the two imaging modalities, they show a moderately positive correlation as shown in Table 2.

Compared to LLRs, the CT values show a higher mean LDFA and lower mean MPTA (both indicating an increased varus alignment). The CT readings also show a more negative aHKA, which denotes an increase in constitutional varus, as well as in joint line obliquity.

### 3.3. Secondary Outcome

Table 3 depicts the difference in distribution of CPAK phenotypes according to both LLR and CT measurements. There was a considerable increase in Types I and IV when utilizing the software-generated readings compared to the LLR measurements.

### 3.4. Intra- and Inter-Observer Reliability of Measurements

The reliability of intra- and inter-observer measurements was assessed using the intraclass correlation coefficient, which demonstrated excellent reliability (score > 0.9). Table 4 illustrates this clearly.

## 4. Discussion

The main finding from our study is that there are statistically significant differences between measurements obtained using LLRs and software-generated CT-based readings, despite a moderately positive correlation (Pearson’s *r* = 0.4–0.7). Nonetheless, the effect sizes for these differences are small (Cohen’s *d* = 0.2) for LDFA, aHKA and JLO, with only MPTA having a moderate effect size (Cohen’s *d* = 0.5). Our results reveal that the determination of coronal lower limb alignment using LLRs underestimates the degree of constitutional varus and joint line obliquity compared to CT scans. When CT scans are utilized, there is a considerable increase in the proportion of patients with CPAK Type I and IV phenotypes (46.8% and 5.0% to 59.8% and 6.6%, respectively, *p* < 0.001).

The most plausible explanation for this would be that CT scans allow the precise 3-dimensional determination of articular weight-bearing points, thus yielding more accurate and consistent coronal measurements [8]. Due to its 2-dimensional nature, LLRs incur the unavoidable risk of error due to flexion and rotational deformities when determining the weight-bearing points. This is further strengthened by the fact that an analysis of our data showed that the mean CT measurements had standard deviations that were 0.52 lower than those of LLR readings for MPTA. Despite this, our good intra- and inter-rater reproducibility scores validate our methodology of measurement using the LLRs and are in keeping with numerous other published studies in literature [5,13,14,15]. 

In their publications, Tarassoli [8] and Ogawa [16] demonstrated that the sagittal position of these landmark points results in significant variation in the resultant coronal alignment obtained. Ogawa published that the “middle” landmark had the best correlation with HKA [16]. A separate study by León-Muñoz comparing LLRs to CT-based 3D models also utilized the “line between the deepest points on the tibial plateau” as their tibial articular axis from which the measurements were derived [11]. These contrast with Tarassoli’s study, which utilized a point two-thirds posterior from the anterior cortex of each plateau (the “66% point”). The authors argue that this point represents the region of greatest stress and should form the basis from which measurements are derived [8]. The Mako SmartRobotics^TM^ System software has been programmed to automatically place the femoral and tibial landmarks at the mid-sagittal point prior to generating alignment parameters [17]. We believe that the mid-sagittal point remains the most relevant, as it represents the most widely utilized landmark. This is largely because weight-bearing LLRs have been the standard of care for guiding pre-op planning of alignment correction prior to TKA surgery, and it would not be possible to adjust these sagittal landmark points on LLRs [8,18].

Various studies in the literature have compared 3D imaging modalities with LLRs, assessing differences between measurements obtained from LLRs against CT scanograms [19,20] and MRI scans performed prior to computer-assisted TKA using patient-specific implants (PSI) [21,22,23]. The primary endpoint in these studies was overall limb alignment or the mHKA. The recently described arithmetic HKA (aHKA) has been demonstrated to be representative of the patient’s constitutional alignment. In that study, the authors utilized LLRs [6]. As the aHKA is dependent only on bony landmarks and is independent of the relationship between the femur and tibia, it can also be measured on non-weight-bearing CT scans. In keeping with the results of our study, Tarassoli showed in his publication that LLRs underestimate the degree of constitutional varus and JLO compared to CT scans [8]. However, in this study, the landmarks are manually configured by the surgeon with assistance from the surgical technicians. In our study, we utilized the newly launched Mako SmartRobotics^TM^ System software, which has been programmed to automatically set these landmarks and generate measurements, pending verification by the trained segmentation specialists [17]. This reduces human error and leverages technology to minimize subjectivity when obtaining alignment measures.

CT-based robotic systems such as the Stryker Mako (Kalamazoo, MI, USA) mandate that a pre-operative CT scan be performed for accurate pre-operative planning. With the rapid adoption of robotic assistance for TKA surgery [24], CT scans are becoming more readily available. The dose of radiation is only approximately 2–3 times more than a complete set of pre-operative radiographs [18,25] and has been deemed to pose an insignificant increment in cancer risk—safe enough for an ongoing large, multi-center randomized controlled trial in the UK [26]. In cases where CT scans are a prerequisite, they could potentially become the default modality to ascertain coronal limb alignment and negate the need for radiographs, including the LLRs. However, in cases that do not necessitate pre-operative CT scans, traditional LLRs remain a valid and time-proven tool for surgical planning.

Another interesting point to note from the results of this study and of that by Tarassoli [8] would be that LLRs underestimate the magnitude of constitutional varus and JLO when compared to CT scans. As highlighted above, despite being the standard of care at present, LLRs are plagued by issues of flexion and rotational deformities and by difficulty identifying the joint line consistently. Both our studies suggest that constitutional varus could be more pronounced than initially imagined, and further larger scale studies should be undertaken to obtain a clearer representation of constitutional alignment, which may influence the alignment strategies undertaken by the surgeon.

This study is not without limitations, which include the fact that this is purely a retrospective comparative radiological study with no consideration of post-operative outcomes. As there is a gradual expansion of evidence regarding the high variability of constitutional alignment across various geographical regions, awareness of this fact and the need to respect patients’ native alignment warrants further research, especially with regard to their post-operative outcomes. Next, this is a single-center study, capturing the demographics and CPAK distribution of an urban Malaysian population. Larger multi-center studies would better capture the demographic distribution. However, the primary objective of this study was to ascertain how closely correlated CT-based software-generated measurements are compared to those we manually measured using LLRs, and we feel that the design of our study accomplishes that. Furthermore, our cohort of 500 knees is the largest in the published literature to our knowledge. Next, we compared supine CT measurements against standing weight-bearing LLRs, which would have been significantly influenced by the effects of loading, but the tibio-femoral relationship is no longer a significant factor as we are comparing aHKA. Subjecting patients to a supine LLR for the purpose of academic comparison incurs unjustified additional radiation and cost.

## 5. Conclusions

Measurements of alignment obtained using LLRs underestimate the degree of constitutional varus and joint line obliquity compared to CT-based software-generated measurements, with a considerable increase in CPAK Types I and IV arising when utilizing the CT measurements. Despite moderately good correlation and a small-to-moderate effect size between both imaging modalities, there remains a statistically significant difference between them.

## Figures and Tables

**Table 1:** Alignment parameters obtained from LLR and CT.

Variable	LLR, ° (Mean ± SD)	CT, ° (Mean ± SD)	Difference, ° (Mean ± SD)	*p* Value ^α^	Cohen’s *d* ^δ^
LDFA	87.76 ± 2.53	88.02 ± 2.88	0.27 ± 2.95	0.045	0.09
MPTA	85.60 ± 2.93	84.45 ± 2.52	1.15 ± 2.20	<0.001	−0.52
aHKA	−2.16 ± 4.26	−3.57 ± 4.30	1.41 ± 3.85	<0.001	−0.37
JLO	173.36 ± 3.44	172.47 ± 3.27	0.89 ± 3.50	<0.001	−0.25

**^α^** Paired sample *t*-test; **^δ^** Cohen’s *d* assessment of effect size: small effect = 0.2, moderate effect = 0.5, large effect = 0.8. LLR: long limb radiographs; CT: computed tomography; LDFA: lateral distal femoral angle; MPTA: medial proximal tibial angle; aHKA: arithmetic hip–knee–ankle axis; JLO: joint line obliquity; SD: standard deviation.

**Table 2:** Assessment of the correlation between measurements obtained from LLR and CT.

Variable	Pearson’s *r*	Sample Size, *N*	*p* Value ^β^
LDFA	0.409	500	<0.001
MPTA	0.683	500	<0.001
aHKA	0.595	500	<0.001
JLO	0.456	500	<0.001

**^β^** Pearson’s Correlation Coefficient Test. Strength of association: weak = 0.1–0.3, moderate = 0.4–0.7, strong ≥ 0.8. LLR: long limb radiographs; CT: computed tomography; LDFA: lateral distal femoral angle; MPTA: medial proximal tibial angle; aHKA: arithmetic hip–knee–ankle axis; JLO: joint line obliquity.

**Table 3:** Distribution of CPAK phenotypes based on LLR and CT measurements.

CPAK Phenotype	LLR % (*n*)	CT % (*n*)	χ^2^ (*df*)	*p* Value
Apex distal JLO				
Type I	46.8 (234)	59.8 (299)	353.07 (4)	<0.001
Type II	25.2 (126)	23.6 (118)		
Type III	13.8 (69)	8.6 (43)		
Proportion of total (*n* = 500)	85.8 (429)	92.0 (460)		
Neutral JLO				
Type IV	5.0 (25)	6.6 (33)	11.80 (2)	<0.001
Type V	7.2 (36)	1.0 (5)		
Type VI	1.8 (9)	0.2 (1)		
Proportion of total (*n* = 500)	14.0 (70)	7.8 (39)		
Apex proximal JLO				
Type VII	0.2 (1)	0.2 (1)	N/A	N/A
Type VIII	0	0		
Type IX	0	0		
Proportion of total (*n* = 500)	0.2 (1)	0.2 (1)		

CPAK: Coronal Plane Alignment of the Knee classification; LLR: long limb radiographs; CT: computed tomography; χ^2^: chi-squared; df: degrees of freedom; JLO: joint line obliquity; N/A: not applicable.

**Table 4:** Assessment of intra- and inter-observer reliability of measurements.

Parameter	Rater	Intraclass Correlation Coefficient							
		Intra-Observer	95% Confidence Interval		Significance, *p*	Inter-Observer	95% Confidence Interval		Significance, *p*
			Lower Bound	Upper Bound			Lower Bound	Upper Bound	
LDFA	1	0.984	0.972	0.992	<0.001	0.991	0.982	0.995	<0.001
	2	0.993	0.986	0.996	<0.001				
	3	0.984	0.970	0.992	<0.001				
MPTA	1	0.980	0.963	0.990	<0.001	0.984	0.970	0.992	<0.001
	2	0.986	0.973	0.993	<0.001				
	3	0.978	0.959	0.989	<0.001				

Two-way mixed effects model, where human effects are random, and measurement effects are fixed: absolute agreement. LDFA: lateral distal femoral angle; MPTA: medial proximal tibial angle.

## Data Availability

The data that support the findings of this study are available on reasonable request from the corresponding author.

## Abbreviations

The following abbreviations are used in this manuscript:

CT	Computed tomography
RATKA	Robotic-assisted Total Knee Arthroplasty
aHKA	Arithmetic hip–knee–ankle axis
JLO	Joint line obliquity
CPAK	Coronal Plane Alignment of the Knee
LDFA	Lateral distal femoral angle
MPTA	Medial proximal tibial angle
TKA	Total Knee Arthroplasty
JLCA	Joint line convergence angle
LLR	Long limb radiographs
3D	Three-dimensional
